# Interim implementation and effectiveness results from the IMplementation of Physical Activity for Children and adolescents on Treatment (IMPACT) intervention and trial

**DOI:** 10.3389/fped.2026.1756594

**Published:** 2026-02-19

**Authors:** Emma McLaughlin, S. Nicole Culos-Reed, Carolina Chamorro-Viña, Beverly Wilson, Sara Fisher, Gregory M. T. Guilcher, Bridget Penney, Mira Penney, Laura Wich, Janine Wich, Colleen Cuthbert, Amanda Wurz

**Affiliations:** 1 Faculty of Kinesiology, University of Calgary, Calgary, AB, Canada; 2 Cumming School of Medicine, University of Calgary, Calgary, AB, Canada; 3 Department of Oncology, Cumming School of Medicine, University of Calgary, Calgary, AB, Canada; 4 Department of Psychosocial Resources, Arthur J.E. Child Comprehensive Cancer Centre, Alberta Health Services, Calgary, AB, Canada; 5 Kids Cancer Care Foundation of Alberta, Calgary, AB, Canada; 6 Department of Pediatrics, Faculty of Medicine and Dentistry, University of Alberta, Edmonton, AB, Canada; 7 Stollery Children’s Hospital, Edmonton, AB, Canada; 8 Hematology/Oncology/BMT Clinic, Alberta Children’s Hospital, Calgary, AB, Canada; 9 Participant Advisory Board, University of Calgary, Calgary, AB, Canada; 10 Faculty of Nursing, University of Calgary, Calgary, AB, Canada; 11 Department of Community Health Sciences, University of Calgary, Calgary, AB, Canada; 12 School of Kinesiology, University of the Fraser Valley, Chilliwack, BC, Canada; 13 BC Children’s Hospital Research Institute, Vancouver, BC, Canada

**Keywords:** effectiveness, exercise, implementation, interim, internet-based, movement, online, pediatric oncology

## Abstract

**Background:**

To support physical activity (PA) among pediatric cancer patients, the IMPACT (IMplementation of Physical Activity for Children and adolescents on Treatment) PA intervention was developed. IMPACT is a 1:1, supervised, PA intervention delivered by exercise professionals over videoconference. It is being evaluated in a hybrid effectiveness–implementation trial. This interim report: (1) examines implementation, (2) explores changes in select secondary effectiveness outcomes, and (3) reviews quality improvement data and documents refinements to date.

**Methods:**

Children and adolescents affected by cancer and blood disorders (5–18 years old), awaiting, on-, or <3 months off-treatment are referred or self-referred to the IMPACT intervention and trial. IMPACT is a 12-week, 1:1, supervised, PA intervention delivered by videoconference by a trained exercise professional. Interim IMPACT implementation covers *reach* (referral rates, participation rate, participant demographics), *adoption* (sources of referrals, difference in referrals across referring sites), and *implementation* (trial retention, adherence to PA sessions, percentage of missing data, intervention delivery time, expertise, PA session fidelity, trial delivery time, adverse events) metrics collected throughout trial delivery*.* Interim *effectiveness* data includes a subset of secondary effectiveness outcomes (quality of life via PedsQL, physical fitness) collected pre- and post-intervention. Additional quality improvement cycle data were collated and reviewed every 6 months. All data were analyzed with descriptive statistics and individual change scores.

**Results:**

Between 1 March 2022 and 12 December 2024, 93 patients were referred (84 = healthcare provider referral; 9 = self-referred), 36 expressed interest, 14 consented and enrolled, and 12 completed the intervention (participation rate = 39%). Retention to the trial was 33%, adherence to PA sessions was 57%, no adverse events were reported, and missing data was 54%. Visual analysis of individual change scores suggests no significant changes in select secondary outcomes. Over 300 intervention and trial delivery hours were accrued, and intervention delivery fidelity was high (95.2 ± 3.83%). Data from quality improvement cycles informed refined and novel recruitment and outreach resources, including posters, brochures, videos, and presentations.

**Conclusions:**

Although levels of referral are high, participation, retention, and adherence rates are low. Results highlight critical areas for improvement to facilitate enrollment, improve adherence, and support data collection for the remaining months of intervention and trial delivery.

## 1 Introduction

Physical activity (PA) during treatment for children and adolescents affected by cancer (i.e., pediatric cancer patients; cancers diagnosed between at <18 years) is feasible, safe, beneficial, and recommended (1–6). Reviews have underscored both the feasibility and safety of PA interventions, with few, if any, adverse events typically reported (1, 3, 7). Further, PA has been found to mitigate symptoms and treatment-related side-effects (e.g., pain, fatigue, and nausea) and improve outcomes across a range of domains, including physical, psychological, social, and cognitive (1, 3, 7–15). Given the evidence to date and the important role of PA for this population, the international Pediatric Oncology Exercise Guidelines were published, suggesting that all children and adolescents affected by cancer (i.e., patients, survivors) *move more* (4) and the Multidisciplinary Network ActiveOncoKids guidelines for providing movement and exercise in pediatric oncology were published reiterating the importance of PA and considerations for implementation and tailoring (5).

Despite this evidence, many children and adolescents during treatment (i.e., pediatric cancer patients) reduce their PA (16, 17) and engage in less PA than their peers without cancer (18). The decrease in PA during treatment may be related to the side-effects described (e.g., pain, nausea, and fatigue), periods of isolation (i.e., in-hospital and at-home), variable treatment timelines (19, 20), and the lack of PA opportunities available (21).

To address these barriers and support PA among pediatric cancer patients during treatment, the IMPACT (IMplementation of Physical Activity for Children and Adolescents on Treatment) intervention was developed and started recruiting from the Alberta Children's Hospital and the Stollery Children's Hospital in 2022. IMPACT includes 1:1 PA sessions delivered by a trained exercise professional over videoconference 3 times/week over 12 weeks and is described further in the Methods section below. IMPACT is currently being evaluated in a type II hybrid effectiveness–implementation trial (22) (see clinical trials registration, accessed on 19 June 2025), and the RE-AIM framework (reach, effectiveness, adoption, implementation, and maintenance (23, 24); is guiding evaluation.

As the IMPACT intervention and trial closed recruitment (31 December 2025), identifying, addressing, and publishing the challenges experienced to date is important. Interim examination of implementation processes and real-world delivery barriers provides an opportunity to understand feasibility and inform refinements while the intervention and trial are ongoing. Moreover, publishing interim implementation results may inform future PA interventions and trials and facilitate transparency in reporting. Thus, the specific objectives of this interim analysis are to: (1) examine implementation over the first 30 months [1 March 2022 (date of trial launch) to 12 December 2024] using the RE-AIM factors of *reach, adoption,* and *implementation*. Reach includes referral rates, participation rate, participant demographics. *Adoption* covers sources of referrals, difference in referrals across referring sites. *Implementation* includes indices of feasibility (trial retention, adherence to PA sessions, percentage of missing data), intervention delivery time, expertise, PA session fidelity, trial delivery time, adverse events; (2) explore changes in select secondary *effectiveness* outcomes of participant-reported quality of life and physical fitness (aerobic endurance, lower body flexibility, shoulder flexion range of motion, balance, and functional mobility); and (3) review quality improvement data and document refinements to date (to support implementation optimization).

## 2 Methods

### 2.1 Study design

An interim analysis of the ongoing type II hybrid effectiveness–implementation trial evaluating IMPACT was conducted. Ethics approval for the full trial was granted from the Research Ethics Boards at the University of Calgary (HREBA.CC-20-0364) and the University of the Fraser Valley (HREB-101287), and administrative approvals were obtained from the children's hospitals in Alberta (the Alberta Children's Hospital and the Stollery Children's Hospital). A detailed overview of all aspects of the PA intervention (IMPACT) and trial has been published (22) and is available at the clinical trials registration (accessed on 19 June 2025). Only methods and analyses pertinent to this interim report are provided herein. Reporting follows guidance from the Consolidated Standards of Reporting Trials (CONSORTs) for eHealth interventions (25) and the Standard for Reporting Implementation Studies (StaRI) statement (26) (see Supplementary File 1).

### 2.2 Participants and recruitment

Potential participants are recruited through referral from healthcare providers at the Alberta Children's Hospital or the Stollery Children's Hospital via consent to contact forms, posters, and self-referral documents, emails from a local support organization (i.e., Kids Cancer Care Foundation of Alberta), and word of mouth.

Eligibility criteria include the following: (1) being a child or adolescent between the ages of 5–18 years at enrollment, (2) having been diagnosed with any cancer or blood disorder, (3) scheduled to receive, currently receiving, or recently completed treatment <3 months ago, (4) being medically cleared to participate in PA, (5) having a caregiver willing to complete assessments and be present during PA sessions, and (6) having capacity to provide informed consent and understand intervention and trial information in English. Of note, the decision to include pediatric blood disorder patients was made considering that pediatric cancer and blood disorder patients are typically treated on the same unit/ward, the supervised, tailored, and low-risk nature of the PA delivered in IMPACT, early available evidence suggesting safety and benefits of PA in this cohort (1–9), and consultations with trial team members who supported including pediatric blood disorder patients).

Interested and eligible participants and their caregivers are sent a link to online consent/assent forms by a member of the trial team via Research Electronic Capture [REDCap; a secure, online, data-capture system (27, 28)]. Participants complete informed consent or assent (as appropriate; see below) and caregivers provide informed consent for themselves (and where required, provide informed consent on behalf of their child). Of note, guidance from Alberta Health Services is being followed to determine whether or not a participant is considered a mature minor, accessed on 19 June 2025; minors typically 14 years and older who can understand and appreciate the nature, risks, and consequence of participating in proposed procedures and who are able to provide consent without the input of their legal guardian. If determined to be a mature minor, the participant provides consent for themself. If determined not to be a mature minor, the participant provides assent for themselves and caregivers complete parental informed consent on behalf of their child.

### 2.3 Intervention

Briefly, the PA intervention (IMPACT) is comprised of tailored PA sessions that are delivered 1:1 by an exercise professional by videoconference (i.e., Zoom) up to 3 times/week for 15–45 min/session, over 12 weeks. Exercise professionals who deliver have completed a comprehensive training, including educational, practical, and trial-specific training [full training details published elsewhere (29)]. Sessions include a combination of aerobic, resistance, balance, core, and flexibility exercises following a detailed protocol. As such, the intervention aligns with the American College of Sports Medicine (ACSM) and Exercise and Sport Science Australia (ESSA) consensus statement on PA and exercise terminology (30), the term PA is used throughout to enhance inclusivity and accessibility. All components (i.e., frequency, intensity, time, and type) are tailored for each participant based on their needs, preferences, and abilities, including how they are feeling and managing treatment-related side-effects, their treatment schedules, and any other considerations (i.e., other commitments). Finally, PA sessions are augmented with behaviour change support via discussion of pertinent and individualized topics (i.e., goal setting, feedback and monitoring, social support, and self-monitoring).

Given PA delivery is by videoconference, several precautions are taken to enhance safety, including obtaining medical clearance for participants and collecting pertinent personal and medical information and consulting with participants and their caregivers, the participants’ healthcare team, and an exercise professional with >20 years of experience in delivering PA for this population to support tailoring PA sessions. Additionally, a detailed protocol (that takes into account the necessity of tailoring) was developed to support safe PA delivery, detailed emergency protocols are available, and caregivers are required to be present during PA sessions (either in the same room or home as the child; depending on age/preferences). With regards to assessments of physical function (see *Effectiveness* below), similar safety precautions are employed.

### 2.4 Data collection

The RE-AIM framework [reach, effectiveness, adoption, implementation, and maintenance; (23, 24)] is guiding evaluation for the trial and this interim analysis. Specifically, selected data related to *reach, effectiveness, adoption*, and *implementation* are reported herein. Data for reach, adoption, and implementation are collected throughout the trial. Effectiveness data are collected at baseline (week 0) and post-intervention (week 12). Data pertaining to quality improvement cycles are collected throughout the trial and are reviewed every 6 months.

#### 2.4.1 Reach (baseline)

The number and characteristics of individuals who participate in IMPACT and the trial is being assessed via referral rate (numbers of referrals), participation rate (number of patients consenting to participate divided by the number of referred individuals overall), and participant characteristics collected at baseline as part of participant- and caregiver-reported questionnaires completed via REDCap, gathering information on personal and medical information, including sex, gender, age, cancer/blood disorder diagnosis, sexual orientation, race/ethnicity, cultural background, and socio-economic status.

#### 2.4.2 Effectiveness (baseline and post-intervention)

Effectiveness is defined as the impact of the intervention on outcomes as measured by participant-reported outcomes and participants' assessments of physical function. Participants' quality of life is assessed via REDCap using the Pediatric Quality of Life Inventory 4.0 Generic Core Scales and PedsQL 3.0 Cancer Module to capture a comprehensive view of health-related quality of life, assessing both general functioning and cancer-specific challenges (31, 32). Assessments of physical function cover participants' aerobic endurance via the 2 min step test (33), lower body flexibility via the sit and reach test (34), shoulder flexion range of motion via the shoulder flexion test (35, 36), balance via the flamingo balance test (37), and functional mobility via the 30 s sit-to-stand test (37) and the timed up and go test (38), which are collected by two exercise professionals over videoconference (i.e., Zoom).

#### 2.4.3 Adoption (throughout)

Adoption is the extent to which settings, organizations, or individuals are willing to start or implement the intervention and trial is being assessed by referral sources, including the number of and types of healthcare providers who refer at each site. In addition, for those who self-refer, the sources through which they learned about IMPACT, including from cancer support organizations, posters and brochures at hospitals, online/social media, word of mouth from other participants, and other sources, are being collected.

#### 2.4.4 Implementation (throughout)

Implementation is how the intervention is being delivered, includes measurements of feasibility including trial retention (i.e., completion of assessments), adherence to PA sessions (i.e., the number of PA sessions attended out of the number of PA sessions offered), and percentage of missing data (i.e., participant-reported outcomes of quality of life and participants' assessments of physical function). Additional implementation markers include intervention delivery time, expertise (i.e., training of qualified exercise professions and assessors), and fidelity (i.e., the assessment of intervention delivery of content as intended following a checklist comprised of 19 items pertaining to safety, exercise tailoring and modifications, and following protocol; a random 10% of PA sessions are assessed by two members of the trial team), trial delivery time (i.e., administrative time), and adverse events during assessments and PA sessions.

#### 2.4.5 Quality improvement cycles (throughout)

Quality improvement cycles explore ongoing intervention delivery and trial conduct and take place every 6 months. During quality improvement cycles, the trial team reviews participants' and caregivers' personal and medical information and intervention fidelity data to identify potential needs for additional recruitment strategies to further diversify the sample or additional training to better support exercise professionals. Only changes that support implementation (e.g., recruitment strategies, training) are considered. At select quality improvement cycles, as deemed necessary by the trial team, interviews are completed with a purposefully recruited subset of healthcare providers and exercise professionals involved with intervention delivery and trial implementation. Interview guides were informed by the Capability, Opportunity, Motivation, and Behaviour (COM-B) framework; (39) and include questions to understand if additional resources are required to support recruitment, referrals, and/or delivery. Interviews are conducted by trained members of the trial team who are not involved in intervention delivery or trial implementation, and audio-recorded and transcribed verbatim.

### 2.5 Participant and caregiver involvement

An advisory board was formally created 12 months into the trial on 25 August 2023 to address the noted limitation of not having patient or caregiver perspectives included in the development of the IMPACT intervention and trial. Currently, the advisory board is comprised of one past participant (of note, initially two past participants comprised the board, however, one passed away), two caregivers, one healthcare provider, one community partner, and four trial team members (including the two co-principal investigators and two coordinators). The advisory board meets quarterly by videoconference to discuss data collected for quality improvement (see *quality improvement cycles* above), to provide advice regarding potential challenges, gaps, and opportunities within the PA intervention and trial implementation, and dissemination of findings. Meetings last 30–60 min. To date, advisory board members have contributed to outputs including news releases [i.e., University of Calgary UToday Article^1^ (accessed on 19 June 2025), Global News^2^ (accessed on 19 June 2025) interview], poster and oral presentations at local, national, and international conferences, and a peer-reviewed publication (22).

### 2.6 Sample size estimation

No sample size was computed given interim report objectives to report data to date and given impending trial cessation. Of note, the sample size estimation for the full trial was set to 76 for primary effectiveness outcomes of PA, and further details are available at the clinical trials registration^3^ (accessed on 19 June 2025) and published protocol (22).

### 2.7 Data analysis

Data covering reach, adoption, and implementation were managed and analyzed in Microsoft Excel version 16.77.1. First, descriptive statistics [i.e., means, standard deviations (SD), numbers, and percentages] were computed to describe data on referral and participation rates, personal and medical information of participants and caregivers, the number and type of healthcare providers who referred between the two sites, trial retention, adherence to PA sessions, percentage of missing data, intervention delivery time and expertise, PA session fidelity, measures of trial delivery time, adverse events, and quality improvement cycles meetings.

With regards to data covering select secondary effectiveness outcomes, the initial intent for this interim analysis was to explore changes from baseline (week 0) to post-intervention (week 12) via Wilcoxon signed-rank tests and report within-group effect sizes. However, given the smaller-than-anticipated sample size and 54% missing data, which was missing not at random (40), the decision was made to modify the data analysis plan and conduct case-by-case analysis and compute individual change scores for participant-reported outcomes of quality of life and assessments of physical function. This aligns with guidance for studies with small sample sizes and high amounts of missing data (41, 42).Finally, quality improvement cycle data for 6 cycles included descriptive data from participants' personal and medical information (analyzed via descriptive statistics as described above) and semi-structured interviews with healthcare providers and exercise professionals (which were briefly read, not analyzed). These data were collated, reviewed, and discussed amongst the trial team. Following trial team review and reflection, advisory board members reviewed and addressed key questions and issues prepared by the trial team. Through discussion and (re-)review of the summarized data, decisions were mutually agreed upon about the development of additional resources to support implementation. Once resources were identified as needed, the trial team led the development of the resources and shared them with the advisory board for input/edits. Herein, the focus of results is on the outputs (i.e., refinements made to date) from the quality improvement cycles. Of note, all refinements have been carefully considered and made only as related to recruitment, supporting exercise professionals conducting assessments of physical function and delivering PA sessions, and not other aspects of the intervention or trial that could influence effectiveness data.

## 3 Results

### 3.1 Reach

**Figure 1:**
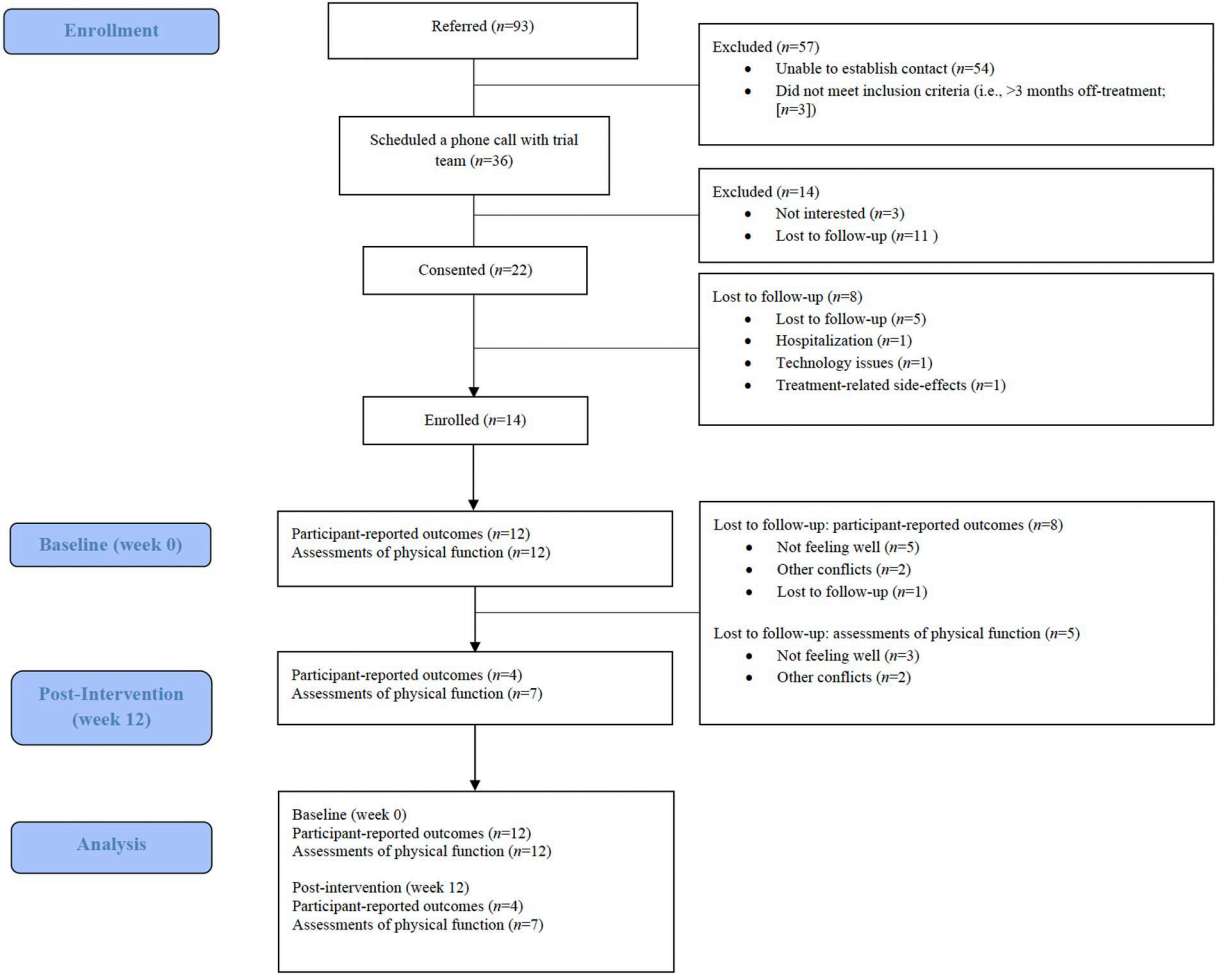
CONSORT diagram, flow of participants.

Between 1 March 2022 (date of trial launch) and 12 December 2024, 93 patients were referred (healthcare provider referral; *n* = 84; 90%) or self-referred (*n* = 9; 10%). Of the 93 participants who were referred, 36 (39%) scheduled a phone call with the trial team to receive more information, 22 (61%) provided informed consent, and 14 enrolled in the intervention for a 39% participation rate. Figure 1 depicts the flow of participants through the intervention and reasons for not participating. At the time of this interim analysis, 12 of the 14 (86%) patients who consented and enrolled completed the intervention, and 2 (14%) are currently in the intervention.

Of the 14 participants (9.69 ± 3.50, 5–16 years old) who consented and enrolled, 6 (43%) identified as female. Participants were of varying ethnicities, with most identifying as South Asian (*n* = 7; 50%), Southeast Asian (*n* = 4; 29%), and White—North American (*n* = 3; 21%). Twelve (86%) participants had cancer, of which varied diagnoses were reported, though acute lymphoblastic leukemia was most commonly reported (*n* = 7; 50%). Two participants were diagnosed with a blood disorder (aplastic anemia; *n* = 2; 14%). Participants were diagnosed with their cancer or blood disorder between 2020 and 2024, and all (100%) were on treatment. Three (21%) participants were treated at the Alberta Children's Hospital, and eleven (79%) were treated at the Stollery Children's Hospital. Table 1 provides further participant details. As mentioned above, two participants are currently completing the intervention.

**Table 1:** Participant demographic and medical characteristics.

Demographic and medical characteristics	Participants (*n* = 14) (%)
Personal factors	
Age (years)	9.69 ± 3.50 (5–16)
Current residence	
Urban location (defined as >100,000 people)	12 (86%)
Rural/remote location (defined as all non-urban locations)	02 (14%)
Biological sex (*n*)	
Female	06 (43%)
Male	08 (57%)
Current gender (*n*)	
Female	06 (43%)
Male	08 (57%)
Ethnicity (*n*)^a^	
Indigenous (Metis, Inuk/Inuit, First Nations, other)	02 (14%)
Southeast Asian (Cambodian, Filipino, Indonesian, Thai, Vietnamese, other)	04 (29%)
South Asian (e.g., Indian, Pakistani, Sri Lankan)	07 (50%)
East Asian (Chinese, Japanese, Korean, Taiwanese descent)	02 (14%)
Black—African (e.g., Ghanaian, Kenyan, Somali)	02 (14%)
White—European (e.g., English, Italian, Portuguese, Russian)	01 (7%)
White—North American (e.g., Canadian, American)	03 (21%)
Medical factors	
Diagnosis (*n*)	
Acute lymphoblastic leukemia	08 (57%)
Aplastic anemia	02 (14%)
Atypical teratoid/rhabdoid tumor	01 (7%)
Burkitt's lymphoma	01 (7%)
Medulloblastoma	01 (7%)
Pilocytic astrocytoma	01 (7%)
Metastases	
Yes	01 (7%)
No	13 (93%)
Current treatment) (*n*)^a^	
Surgery	03 (21%)
Chemotherapy	12 (86%)
Other	02 (14%)
Completed treatment) (*n*)^a^	
Surgery	05 (36%)
Chemotherapy	07 (50%)
Radiation	01 (7%)
Stem cell transplant	02 (14%)
Other	02 (14%)
Date of diagnosis (month/year)	
07/2020–12/2020	01 (7%)
01/2021–06/2021	01 (7%)
07/2021–12/2021	01 (7%)
01/2022–06/2022	00 (0%)
07/2022–12/2022	04 (29%)
01/2023–06/2023	04 (29%)
07/2023–12/2023	01 (7%)
01/2024–06/2024	01 (7%)
07/2024–12/2024	01 (7%)
Symptoms (*n*)^a^	
Fatigue	12 (86%)
Pain	06 (43%)
Lymphedema (swelling)	01 (7%)
Peripheral neuropathy (tingling, numbness) or other nerve damage	04 (29%)
Muscle or joint issues (e.g., loss of mass, reduced range of motion, pain, stiffness)	08 (57%)
Bladder or bowel problems (incontinence), or issues with pelvic floor weakness	01 (7%)
Cognitive challenges (learning or memory problems, chemo brain, brain fog)	02 (14%)
Weight maintenance issues	03 (21%)
Other	02 (14%)
Previously participated in physical activity	
Yes	11 (79%)
No	03 (21%)

a Participants could select more than one response; %, percentage.

### 3.2 Effectiveness

Table 2 includes individual change scores for the 12 participants who completed the intervention on outcomes of quality of life and scores on measures assessing aerobic endurance, lower body flexibility, shoulder flexion range of motion, balance, and functional mobility. Complete participant-reported outcome data (i.e., quality of life) were available at baseline for all (*n* = 12; 100%), and scores for quality of life ranged from low to moderate. At post-intervention (week 12), only four (33%) completed participant-reported outcomes, and quality of life scores also ranged from low to high. Looking at those with complete data, general quality of life increased for all, and cancer-specific quality of life increased for one, stayed the same for one, and decreased for two.

**Table 2:** Baseline (week 0) and post-intervention (week 12) individual change scores.

Participant	PedsQL 4.0 generic core	PedsQL 3.0 cancer module	2 min step test	Sit and REACH TEst (cm)		Shoulder flexion range of motion (degrees)		Balance (seconds)		30 s Sit-to-stand test	Timed up and go test (seconds)
				Right	Left	Right	Left	Right	Left		
P01											
Baseline (Week 0)	54.35	44.23	96.00	−0.50	−1.50	175.00	177.50	37.50	45.00	16.00	7.55
Post-intervention (Week 12)	54.36	46.15	89.00	−18.00	−15.00	177.50	170.00	12.90	33.50	21.00	7.90
P04											
Baseline (Week 0)	52.17	57.69	61.00	DNC	DNC	155.00	157.50	1.50	2.50	12.00	9.88
Post-intervention (Week 12)	67.39	57.69	85.00	−13.00	−8.00	130.00	140.00	3.80	2.80	8.00	12.90
P05											
Baseline (Week 0)	70.65	86.11	57.00	−7.50	−13.50	182.50	182.50	14.60	12.50	17.00	8.18
Post-intervention (Week 12)	80.43	82.40	91.00	−15.00	−15.00	180.00	180.00	45.00	45.00	22.00	5.45
P06											
Baseline (Week 0)	52.17	83.93	60.00	−26.00	−20.00	157.50	155.00	20.00	3.60	14.00	8.26
Post-intervention (Week 12)	DNC	DNC	DNC	DNC	DNC	DNC	DNC	DNC	DNC	DNC	DNC
P07											
Baseline (Week 0)	44.57	56.25	DNC	−35.50	−37.00	145.00	155.00	7.60	7.80	DNC	DNC
Post-intervention (Week 12)	51.09	54.63	90.00	−40.00	−40.50	165.00	160.00	2.50	2.00	10.00	9.34
P11											
Baseline (Week 0)	83.70	85.71	86.00	−7.50	−6.50	145.00	152.50	DNC	DNC	DNC	DNC
Post-intervention (Week 12)	DNC	DNC	DNC	DNC	DNC	DNC	DNC	DNC	DNC	DNC	DNC
P14											
Baseline (Week 0)	51.09	68.96	92.00	−15.00	−15.00	167.50	165.00	11.20	11.20	23.00	9.15
Post-intervention (Week 12)	DNC	DNC	79.00	−11.50	−16.50	177.50	170.00	20.40	9.80	29.00	6.51
P15											
Baseline (Week 0)	58.70	59.00	83.00	−12.00	−13.00	157.50	157.50	45.00	45.00	13.00	4.92
Post-intervention (Week 12)	DNC	DNC	100.00	−10.00	−11.00	167.50	167.50	45.00	45.00	14.00	4.67
P19											
Baseline (Week 0)	58.70	82.69	51.00	−15.00	−19.00	145.00	160.00	19.50	6.50	10.00	11.25
Post-intervention (Week 12)	DNC	DNC	39.00	−24.50	−26.50	150.00	145.00	24.10	2.40	4.00	20.80^b^
P21											
Baseline (Week 0)	58.70	59.62	39.00^a^	−12.50^b^	−18.00	180.00	180.00	DNC	5.40	15.00	7.40
Post-intervention (Week 12)	DNC	DNC	DNC	DNC	DNC	DNC	DNC	DNC	DNC	DNC	DNC
P24											
Baseline (Week 0)	70.65	66.18	97.00	−18.00	−15.00	170.00	170.00	37.10	35.00	26.00	5.53
Post-intervention (Week 12)	DNC	DNC	DNC	DNC	DNC	DNC	DNC	DNC	DNC	DNC	DNC
P25											
Baseline (Week 0)	63.04	65.38	63.00	−21.50	−18.00	165.00	170.00	15.40	4.20	12.00	6.62
Post-intervention (Week 12)	DNC	DNC	DNC	DNC	DNC	DNC	DNC	DNC	DNC	DNC	DNC

cm, centimeters; DNC, did not complete.

a Ended assessment early at 55 s.

b Only completed one trial due to reported fatigue.

Complete physical function outcome data (i.e., aerobic endurance, lower body flexibility, shoulder flexion range of motion, balance, and functional mobility) were available for 11 (92%) at baseline and scores ranged from low to moderate on aerobic endurance, low for lower body flexibility, mild restriction to normal shoulder flexion range of motion, low to high on balance, and low to high on functional mobility. At post-intervention (week 12), only seven (58%) participants completed assessments of physical function. Again, scores ranged from low to moderate on aerobic endurance, low for lower body flexibility, mild restriction to normal shoulder flexion range of motion, low to high on balance, and low to high on functional mobility. Looking at those with complete data, aerobic endurance (2 min step test) improved for three and worsened for four. Lower body flexibility right side (sit and reach test) improved for two and worsened for four, whereas lower body flexibility left side improved for one and worsened for five. Shoulder range of motion right side (shoulder flexion test) improved for five and worsened for two, and shoulder range of motion left side improved for three and worsened for four. Right side balance (flamingo balance test) improved for four, stayed the same for one, and worsened for two, whereas left side balance improved for two, stayed the same for one, and worsened for four. Functional mobility via the 30 s sit-to-stand test improved for four and worsened for two, and the timed up and go test improved for three and worsened for three.

### 3.3 Adoption

Eighty-four healthcare provider referrals were made, which were distributed relatively even between the Alberta Children's Hospital (*n* = 35; 42%) and the Stollery Children's Hospital (*n* = 49; 58%). Potential participants who self-referred indicated hearing about the intervention and trial from a cancer support organization (Kids Cancer Care Foundation of Alberta; *n* = 3; 33%) and posters at hospitals (*n* = 6; 67%). Of the 14 participants who consented and enrolled, all (100%) had also heard about the intervention and trial from healthcare providers, specifically from participants' physical therapists (*n* = *9*; 64%), oncologists (*n* = 4; 29%), and nurses (*n* = 2; 14%). All participants from Alberta Children's Hospital (*n* = 3; 100%) were referred by a pediatric oncologist and 9 (82%) from Stollery were referred by a physical therapist, and the remaining were referred by a nurse (*n* = 2; 18%) and pediatric oncologist (*n* = 1; 9%).

### 3.4 Implementation

Of the 14 participants who consented and enrolled, all (100%) completed baseline assessments of participant-reported outcomes of quality of life and physical function. Of the 12 participants who completed the intervention, 4 (33%) completed participant-reported outcomes, and 7 (58%) completed assessments of physical function, resulting in 33% retention in the trial. Reasons for not completing participant-reported outcomes (*n* = 8) included not feeling well (*n* = 5; 63%), other conflicts (*n* = 2; 25%), and lost to follow-up (*n* = 1; 13%). Reasons for not completing assessments of physical function (*n* = 5) included not feeling well (*n* = 3; 60%) and other conflicts (*n* = 2; 40%). Missing data for participant-reported outcomes of quality of life and assessments of physical function were 54%. Of the three PA sessions that were offered to each participant per week, on average, 2 ± 1 sessions (range = 0–3 sessions) were completed each week, resulting in 57% adherence to PA sessions.

For the 12 participants who completed the intervention, intervention delivery and administrative time to date have been >300 h. Across baseline and post-intervention assessments of physical function, 19 (of a possible 24) were conducted, totalling 9.5 h of assessments. Six trained assessors have been involved in conducting these 19 assessments: four graduate students served as primary assessors (i.e., leading the assessments of physical function by talking and demonstrating the various assessments), while six graduate and undergraduate students served as secondary assessors (i.e., supporting the primary assessor by counting and timing). In terms of expertise, all assessors had prior experience conducting assessments of physical function for young adults and/or adults affected by cancer, both in-person and delivered by videoconference (43–45). With regards to training, all assessors completed the Thrive Health Cancer and Exercise training^4^ (accessed on 19 June 2025) and have experience conducting similar videoconference-based assessments with young adults and adults living with and beyond cancer (43–45). Additionally, all assessors received IMPACT-specific training, which included a 30 min review of the assessments with one of the trial coordinators.

Across the 12 participants who completed the intervention, a total of 200 PA sessions have been delivered, totalling >115 delivery hours. These sessions were led by one of three exercise professionals (graduate students) who have expertise and experience in delivering PA classes delivered by videoconference for adults affected by cancer (43, 44). Regarding training, all exercise professionals completed a comprehensive >30 h educational [i.e., Thrive Health Cancer and Exercise training (see text footnote 4) (accessed on 19 June 2025) as well as the pediatric cancer and exercise^5^ (accessed on 19 June 2025) and adolescent and young adult^6^ (accessed on 19 June 2025) specific modules], practical application (i.e., scenario-based competency training session, shadowed an existing group-based PA program in the community [i.e., PEER,^7^ accessed on 19 June 2025; (46)], and trial-specific training (e.g., oncology treatment and side-effects, modifying PA based on treatment-related side-effects, supporting movement, intervention processes).

Trial coordination has been managed by two trial members (a PhD candidate and a staff research administrator), who have collectively worked 215 h. Their responsibilities include outreach (e.g., emails to healthcare providers and cancer support organizations, updating recruitment materials, and scheduling presentations), liaising with healthcare providers, receiving consent to contact forms, communicating with participants and caregivers, administering consent (and assent as appropriate), distributing participant-reported outcome questionnaires, scheduling participants' assessments of physical function, coordinating the primary and secondary assessors, and connecting participants with the exercise professional. Coordinators oversee the training and onboarding of other trial staff, including exercise professionals, primary and secondary assessors, and research assistants.

A fidelity audit was conducted on a random sample of 10% of PA sessions for each participant, with two trial team members completing a 19-item standardized checklist. Fidelity was high (95.2% ± 3.83; 88.9%–100%), and indicated that exercise professionals appropriately welcomed participants, tailored exercises safely and effectively based on participants' needs, energy, preferences, and abilities, and incorporated various behaviour change techniques (e.g., goal setting and self-monitoring) throughout the PA sessions to further support participants. No adverse events were reported by participants, caregivers, or exercise professionals.

### 3.5 Quality improvement cycles

From March 2022 to December 2024, six quality improvement cycles have occurred, three of which included the advisory board. During quality improvement cycle meetings, whether with trial staff only (March 2022 to March 2023) or trial staff and advisory board members (September 2023 to present), collated data identified issues between the number of referrals and the number of participants and caregivers consenting and enrolling. In an effort to support consenting, additional resources (e.g., posters, brochures, a frequently asked questions document; see Supplementary File 2) were iteratively developed and three short (2–3 min) videos were developed to inform potential participants (patients) and caregivers about the IMPACT intervention and trial and the assessments of physical function (see Supplementary File 2). Additional issues were noted with the number of referrals and the need to enhance the number of referring healthcare providers. Thus, four outreach presentations were conducted by the trial team at both hospitals to increase healthcare provider awareness about IMPACT. Finally, issues were noted with regard to assessment completion (see missing data above). Discussions with the advisory board suggested a need for greater scheduling flexibility (i.e., times during the day and evening) for assessments of physical function and PA sessions and scheduling auto-reminders. In response, two additional assessors were trained since the onset of this study to offer greater scheduling flexibility, especially in the evening.

## 4 Discussion

This interim report provides an overview of select implementation (including reach, adoption, and implementation) and secondary effectiveness data from the IMPACT intervention and trial. Findings indicate high referral rates, adoption, and fidelity. However, low participation, retention, and adherence rates and high amounts of missing data suggest critical areas for improvement.

Participation in this trial (39%) is at the low end of what has been published previously with in-person, supervised PA interventions (31%–88%); (47–51), suggesting videoconference-delivered PA interventions may be subject to lower participation than in-person interventions. When comparing participation to other PA interventions delivered by videoconference, our participation was lower than 52.9% reported in an 8-week PA intervention for pediatric cancer survivors delivered 2 times/week for 30 min (with unsupervised sessions 5 times/week for 30 min) (52) and higher than the 21% reported in a 16-week PA intervention for pediatric cancer survivors delivered 2 times/week for 35–45 min (53). Further work is needed to understand these varied participation rates and whether videoconference-delivered PA is needed and wanted by this cohort. Similar to past work, reasons for non-participation covered treatment-related side-effects (e.g., pain, fatigue, and nausea), caregiver burden, and prioritizing treatment (19, 54). Educating patients, caregivers, and healthcare providers and staff (who may play a critical role in supporting/hindering PA) on the safety, benefits, and adaptability of PA (in IMPACT and generally), and integrating this information within recruitment materials, may increase participation rates and could be warranted. Further, the gap in participation rate was seen between referral and enrollment. As such, finding ways to make recruitment playful and fun so that prospective participants and their caregivers see the PA intervention as fun may be one way to better support enrolment.

With respect to effectiveness outcomes, the goal was to explore individual change scores of quality of life and assessments of physical function. Given the small sample size and high percentage of data missing not at random, no meaningful conclusions or inferences can be made about the effectiveness of the PA intervention at this point. Individual change scores revealed mixed findings across participants, with some participants experiencing no change or improvement, and others experiencing worsening in outcomes following the PA intervention. This aligns with past published research exploring PA among pediatric cancer patients (6–8, 55, 56). Indeed, worsened outcomes may reflect the stage of treatment and anticipated adverse impacts. Thus, while PA is feasible, safe, beneficial, and recommended, it is plausible that PA during treatment may not confer significant improvements but rather protect against the commonly observed declines (3, 6, 57, 58). While control groups allow for comparison between groups to better understand the effects of a PA intervention during treatment, PA is considered a basic right for all children and adolescents (59), and withholding access may raise ethical concerns. Current guidelines for pediatric cancer patients and survivors state “*movement may look (and feel) different day to day, and that is okay*” and that PA should be tailored to participants needs, preferences, medical factors (including needs to balance physical exertion with rest), age, and abilities, including how they are feeling and managing treatment-related side-effects, their treatment schedules, and any other considerations [i.e., other commitments; (4)]. Looking ahead, exploring alternate ways to examine intervention effects, such as preference-based interventions or waitlist control groups, may be warranted [and have been recommended to advance the field; (60);]. Further, creative ways to explore changes in outcomes and report on intervention effects, such as moment-to-moment or ecological momentary assessments (61, 62) or using visual analogue scales pre/post PA sessions (62), may also better capture effects from single sessions vs. exploring change over a time wherein declines are expected (i.e., during treatment). Finally, collecting and analyzing qualitative data within mixed methods or purely qualitative studies may help provide insights into benefits experienced by participants (or not). Notably, qualitative data are being collected within the IMPACT trial and will be included in the final report.

With regards to retention in the trial (33%) and adherence to PA sessions (57%), both metrics were lower than previously published studies [i.e., 70%–100% (2, 61)]. Further, a high percentage of missing data (54%) was noted. These rates could signal issues with assessment burden and underscore the challenges implicit with engaging pediatric cancer patients in PA during treatment [e.g., treatment-related side-effects, periods of isolation, variable treatment timelines, and lack of PA opportunities; (19–21)]. These findings could also reflect the lack of patient and caregiver voices included during IMPACT intervention and trial development. Involving patients and caregivers from the outset is critically important when seeking to develop interventions that are needed and wanted, and the rates presented herein may reflect the absence of their perspective earlier in the research process. To the trial team's knowledge, pediatric exercise oncology interventions to date have not described involving patients in the planning and development or dissemination phases of research, despite recognition of the importance of patient-oriented research (62, 63). Researchers in the field have previously suggested engaging end users in defining priority outcomes to ensure they are relevant to participants (60). This limited integration of patient perspectives represents a clear call to action to incorporate patients' voices throughout the entire research process, from designing the study to developing and delivering the intervention, to analyzing and disseminating findings. Doing so may help ensure interventions are appropriate and meaningful and that findings reach intended knowledge users (62, 63) and that metrics such as particiation, retention, and adherence are higher. In planned (and funded) future work to adapt, refine, and reconsider IMPACT (64), an integrated knowledge translation and patient-oriented research approach has been adopted, and patients and caregivers are being engaged at the outset (along with healthcare providers and support organization personnel). Finally, findings also underscore the need to consider ways to enhance data completeness beyond efforts that have already been made (i.e., increasing scheduling flexibility and sending auto-reminders). This may include considering the timing at which assessments are conducted, limiting and focusing outcomes measured to reduce participant burden, and ensuring measures capture outcomes relevant to participants.

With respect to intervention and trial delivery hours, >115 h and 215 h have been completed, respectively. These metrics are rarely reported in pediatric exercise oncology and are important to provide a sense of the time required to deliver the intervention and trial, and to help those planning to conduct similar interventions and trials. Future work will include costing analyses (and estimates) to better understand the economic implications of the intervention and trial. With respect to fidelity, the tailored PA protocol delivered by exercise professionals showed high intervention fidelity (95.2% ± 3.83; 88.9%–100%), indicating exercise professionals can reliably tailor PA sessions based on participants needs, preferences, medical factors, age, and abilities, including how they are feeling and managing treatment-related side-effects, their treatment schedules, and any other considerations (i.e., other commitments). No adverse events have been reported to date, underscoring the potential safety of videoconference-delivered PA when supported by an exercise professional, which is similar to PA delivery in-person (3, 8, 65).

Findings from this interim analysis underscore the less-than-successful aspects of the IMPACT intervention and trial to date and provide critical insight into reach, effectiveness, adoption, implementation, and refinements made to date and highlight essential areas of focus for the remaining time prior to trial cessation. There are also important considerations to take into account when interpreting the findings herein, including the ongoing nature of the trial. As such, results are not definitive. Relatedly, the small sample size, low retention rate, and adherence rate and high percentage of data missing not at random adversely impacted the ability to analyze select secondary effectiveness outcomes as planned for this interim analysis. No meaningful conclusions or inferences can be made about the effectiveness of the PA intervention. Further, the target sample size for the full trial (*n* = 76 participants) and those included within this interim report (*n* = 12) highlights significant implementation challenges, some of which we intend to address to enhance enrolment and participation for the remainder of the trial and others that will be considered in planned (and funded) adaptation work. Any modifications made to optimize implementation will continue to be tracked and reported in forthcoming manuscripts, including the full trial results. Finally, results raise important concerns regarding videoconference delivery and whether this is a viable option for this population. In planned (and funded) future work, patients will be engaged early and as partners to explore whether solely videoconference-delivered PA is suitable.

Taken together, findings provide important insights and underscore areas of focus to inform the remaining months of IMPACT intervention and trial delivery. It is hoped that this interim report provides lessons for others wanting to develop and deliver PA interventions to support this population, and similar other populations. Ultimately, this work contributes to the growing body of literature seeking to help pediatric cancer patients *move more*.

## Data Availability

The datasets presented in this article are not readily available because of ethical reasons. The data presented in this study are available on request from the corresponding author. Requests to access the datasets should be directed to Dr. Amanda Wurz, Amanda.Wurz@ufv.ca.

## Footnotes

1
https://ucalgary.ca/news/ucalgary-studys-innovative-approach-supports-kids-and-families-staying-active-during-cancer-care

2
https://globalnews.ca/news/9956323/calgary-study-children-exercise-cancer-treatment/

3
https://clinicaltrials.gov/study/NCT04956133

4
https://thrivehealthservices.com/professionals/certification/

5
https://thrive-s-school-c9c2.thinkific.com/courses/pediatric-cancer-and-exercise-module

6
https://thrive-s-school-c9c2.thinkific.com/courses/adolescent-and-young-adults-with-cancer-the-role-of-exercise-module

7
https://www.kidscancercare.ab.ca/for-families/camp-and-outreach/
