# Supplementary material for: Interim implementation and effectiveness results from the IMplementation of Physical Activity for Children and adolescents on Treatment (IMPACT) intervention and trial
Source: Front Pediatr. 2026 Feb 19;14:1756594. doi: 10.3389/fped.2026.1756594 (PMC12960623; doi:10.3389/fped.2026.1756594)
Supplement: Supplementary file 2 [file Supplementaryfile2.pdf]

Supplementary File 2. Resources developed from quality improvement cycles.

Poster

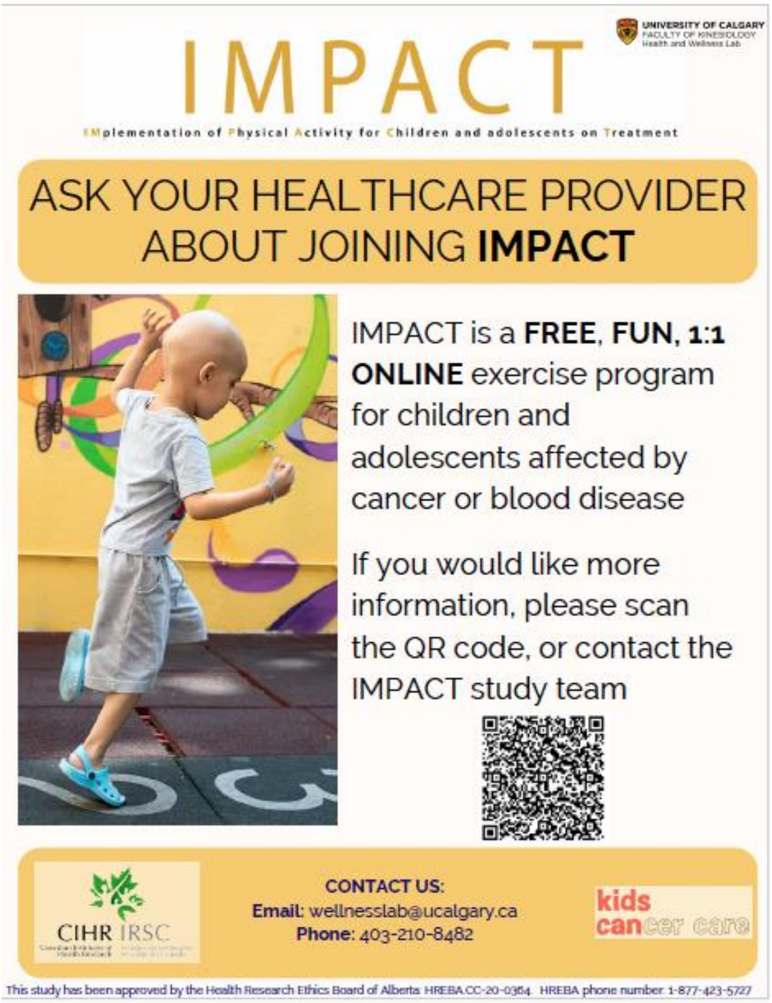

Brochure

Did you know?

Physical activity is **safe and beneficial** for your child

Physical activity can help to:

- Promote health and boost immune systems
- Increase energy
- Enhance mood and confidence
- Support cognitive skills
- Improve physical fitness

IMPACT is funded by:

CIHR IRSC Canadian Institutes of Health Research Instituts de recherche en santé du Canada

UNIVERSITY OF CALGARY FACULTY OF KINESIOLOGY Health and Wellness Lab

Arnie Charbonneau Cancer Institute

kids cancer care

This study has been approved by the Health Research Ethics Board of Alberta: HREBA.CC-20-0364
HREBA phone number: 1-877-423-5727

PEER is funded by:

Foundation

kids cancer care

Physical Activity for Children with Cancer and Blood Disease

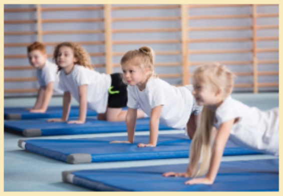

Supporting Your Child to Move More

IMPACT Exercise Study

IMPACT

IMplementation of Physical Activity for Children and adolescents on Treatment

IMPACT is a safe and tailored physical activity program for children and adolescents (5-18 years old) in Alberta who have been diagnosed with any cancer or blood disease that are pre-, on-, or <3 months off-treatment.

The IMPACT program offers 1-on-1 sessions with a qualified exercise professional 3 times/week for 12 weeks. Sessions range from 15-45 minutes.

To learn more about the IMPACT program, scan the QR code for contact us by phone or email.

Contact Us:

wellnesslab@ucalgary.ca

(403) 210-8482

Join us now in moving more!

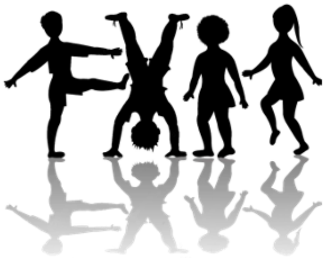

Interested in adding more movement into your routine?

Contact IMPACT or PEER today!

PEER Program

PEER

The PEER program at Kids Cancer Care is an inclusive, fun, safe, and free physical activity program designed to reduce side-effects of cancer and its treatment, boost energy and develop physical literacy.

PEER offers group sessions and 1-on-1 sessions for kids on and off treatment and their siblings (2-17 years old).

To learn more about the PEER program, scan the QR code or contact us by phone or email.

Contact Us:

cchamorro@kidscancer.ab.ca

(403) 984 - 1218

Frequently asked questions document

IMPACT Frequently Asked Questions

Who is the IMPACT program for?

IMPACT is for children and adolescents who are:

- 5 to 18 years old.
- Have a cancer or blood disease diagnosis.
- Pre-treatment, on-treatment, or <3 months off-treatment.

Where do the IMPACT sessions take place?

All IMPACT sessions are conducted online via Zoom.

What is the time commitment?

Participation includes:

- Child and caregiver completing consent/assent (approx. 5-10 minutes).
- Child and caregiver completing online questionnaires (approx. 30-45 minutes) at multiple timepoints (week 0, week 12, week 24, 1 year).
- Child completing an online fitness assessment (approx.20-30 minutes) at multiple timepoints (week 0, week 12, week 24, 1 year).
- Child completing 12 weeks of 2-3 weekly physical activity sessions with a qualified exercise professional.
  - Each session lasting 15-45 minutes. The study team is flexible to meet the number of days/times that work best for you and your child.

Who leads the sessions?

The physical activity sessions are led by qualified trained exercise professionals with exercise oncology training, teaching experience, pediatric oncology and blood disease knowledge and training.

What role does the caregiver or guardian have in the IMPACT program?

As the caregiver, we ask that you or another adult be present during the online fitness assessments and the physical activity sessions for safety purposes.

What is involved with the IMPACT fitness assessments?

For the fitness assessment your child is encouraged to wear comfortable clothing they can move in. You will need a chair where your child's knees bend at 90 degrees, a measuring tape or a ruler and an object to measure a distance of 3 meters or 10 feet.

***Tests will only be performed if your child feels comfortable with the movements. The tests can be stopped at any time and breaks can be taken throughout.**

There are 6 tests included in the assessment:

- 2-minute step test, sit and reach, single leg balance, shoulder range of motion, timed up and go, and 30-second sit to stand.

Scan the QR code to see what our fitness assessments look like! **Children :** **Teens :**

What if my child is not feeling well?

If your child is feeling tired, they are still welcome to join the physical activity session and the qualified exercise professional will modify the movements and session length based on how your child is feeling. This might include lower intensity movements like stretching.

How do I sign my child up for IMPACT?

You can contact the IMPACT study team at wellnesslab@ucalgary.ca or call 403-210-8482 for more information. A member of the study team will be in contact with you in 1-2 business days.

What if I have more questions?

You can contact the IMPACT study team through email at wellnesslab@ucalgary.ca or call 403-210-8482.

kids cancer care · CIHR IRSC · Arnie Charbonneau Cancer Institute · University of Calgary Faculty of Kinesiology Health and Wellness Lab

Videos:

- IMPACT overview: https://youtu.be/LZ9EjZH8eYY?si=hxw_xhq_xP3v3OXD
- Physical function overview – Children: https://youtu.be/aWvY02G2DbQ?si=jBZdSlwY9TaRUPMK
- Physical function overview – Adolescents: https://youtu.be/qEpdNN9KTmA?si=aXi3dr1Z9CKldNG-
